# Exploring the prospective of weeds (*Cannabis sativa* L.*, Parthenium hysterophorus* L.) for biofuel production through nanocatalytic (Co, Ni) gasification

**DOI:** 10.1186/s13068-020-01785-x

**Published:** 2020-08-20

**Authors:** Nadeem Tahir, Muhammad Naveed Tahir, Mujeeb Alam, Wang Yi, Quangou Zhang

**Affiliations:** 1 Collaborative Innovation Center of Biomass Energy, Henan Agricultural University, Zhengzhou, 450002 China; 2 Department of Agronomy, PMAS-Arid Agriculture University Rawalpindi, Rawalpindi, 46300 Pakistan

**Keywords:** Weeds, Nano-catalytic gasification, Biofuel, Biomass, Biochar

## Abstract

**Background:**

While keeping in view various aspects of energy demand, quest for the renewable energy sources is utmost. Biomass has shown great potential as green energy source with supply of approximately 14% of world total energy demand, and great source of carbon capture. It is abundant in various forms including agricultural, forestry residues, and unwanted plants (weeds). The rapid growth of weeds not only affects the yield of the crop, but also has strong consequences on the environment. These weeds can grow with minimum nutrient input requirements, have strong ability to grow at various soil and climate environments with high value of cellulose, thus can be valuable source of energy production.

**Results:**

*Parthenium hysterophorus* L. and *Cannabis sativa* L. have been employed for the production of biofuels (biogas, biodiesel and biochar) through nano-catalytic gasification by employing Co and Ni as nanocatalysts. Nanocatalysts were synthesized through well-established sol–gel method. SEM study confirms the spherical morphology of the nanocatalysts with size distribution of 20–50 nm. XRD measurements reveal that fabricated nanocatalysts have pure standard crystal structure without impurity. During gasification of *Cannabis sativa* L., we have extracted the 53.33% of oil, 34.66% of biochar and 12% gas whereas in the case of *Parthenium hysterophorus* L. 44% oil, 38.36% biochar and 17.66% of gas was measured. Electrical conductivity in biochar of *Cannabis sativa* L. and *Parthenium hysterophorus* L. was observed 0.4 dSm−1 and 0.39 dSm−1, respectively.

**Conclusion:**

Present study presents the conversion of unwanted plants *Parthenium hysterophorus* L. and *Cannabis sativa* L. weeds to biofuels. Nanocatalysts help to enhance the conversion of biomass to biofuel due to large surface reactivity. Our findings suggest potential utilization of unwanted plants for biofuel production, which can help to share the burden of energy demand. Biochar produced during gasification can replace chemical fertilizers for soil remediation and to enhance the crop productivity.

## Background

Economies around the world are facing serious threats because of high energy demands for sustainable economic growth and development. While keeping many challenges in view such as limited resources, high prices of conventional fuels, and environmental pollution, research and development in the renewable energy sources is utmost solution [1, 2]. Biomass has shown great potential as green energy source with supply of approximately 14% of world total energy demand, and great source of carbon capture [3]. It is abundant in various forms including agricultural, forestry residues, and unwanted plants (weeds). Feedstocks for second-generation biofuels are already being promoted and include such plant species which can grow fast with minimum nutrient input requirements, have strong ability to grow at various soil and climate environments with high value of cellulose. Most of the work is carried out by adopting biological methods to produce biofuel from various species including *Salix* spp. [4, 5], Eucalyptus spp. [6], Prosopis spp. [7], *Parthenium hysterophorus* L. [8], *Cannabis sativa* L. [9], *Panicum virgatum* L. [10, 11] and Arundo donax L. [12].

The pyrolysis, liquefaction, combustion and gasification are the fundamental thermochemical conversion routes of biomass to biofuel which end up with bioethanol, biodiesel, bio-oil, bio-syngas and biohydrogen. Catalytic gasification has shown great advancement in production of clean energy by converting biomass at low gasification temperatures with high efficiency. The employed catalysts not only help to reduce the reaction time but also help to lower the conversion temperature of biomass to gas of high calorific value [13, 14].

Nanocatalysts have shown great potential to overcome limitations barrier faced by conventional catalysts due to entirely different properties as compared to bulk materials with high surface reactivity. Various nanomaterials have been employed as catalysts including metallic nanoparticles, nanotubes, and nanorods in numerous applications for the production of bioethanol and biodiesel [15, 16]. Catalysts play crucial role in production of biodiesel where transesterification of fats, vegetable oils, and grease (FOG) is carried out through the addition of methanol (or other alcohols). In the standard procedure, the production of biodiesel from biomass is carried out through two steps. During the first step, the biomass is gasified at elevated temperature with resultant byproduct of bio-oil, syngas and biochar which can be good alternative to chemical fertilizers. In the second step, bio-oil is further passed through transesterification to get biodiesel [17]. Biodiesel derived from biomass has been promoted under the aspect of being a ‘‘premium’’ diesel fuel with, for example, a very high cetane number [18].

Addition of catalyst enhances the reaction rate of transesterification process and aids in producing high yields of biodiesel [19]. In relation to substrate phase, they are categorized into homogenous and heterogeneous catalysts [20]. Heterogeneous catalysts have several advantages including ease of recycling by filtration which reduces the operational cost by reusability. Various metallic oxides have been examined for the transesterification process of oils and have emerged as potential heterogeneous catalysts; these include alkali earth metal oxides, transition metal oxides, mixed metal oxides, and supported metal oxides [21]. Veljkovic et al. [22] studied the kinetic of calcined CaO at 500 °C for the transesterification process of biodiesel production from sunflower oil. The reaction was performed using 6:1 mol ratio of methanol to oil, 2 h reaction time, 1 wt% catalyst and 60 °C to achieve 98% of FAME yield. Zhao et al. [23] carried out transesterification of canola oil and methanol using a batch reactor at optimum conditions of 65 °C, with methanol to oil molar ratio of 9:1 and 600 rpm stirring speed. The biodiesel yield over nano-CaO was nearly 81%. Taufiq-Yap et al. [24] showed that by employing metal oxides for catalyzing the transesterification reaction of non-edible *Jatropha curcas* oil to produce biodiesel can be possible route to achieve good results. The catalyst with optimum reaction conditions; 25:1 M ratio of methanol to oil, 3 h, 120 °C, and catalyst loading of 3 wt % for various Ca/Mg atomic ratios show FAME with 70–90% yield range. Safdar Ali et al. [25] showed the production of biogas, biodiesel and biochar from *Carthamus oxyacantha*, *Asphodelus tenuifolius* and *Chenopodium album* through nano-catalytic gasification by employing Ni and Co nanocatalysts, where biodiesel contained 65.47% esters contents. It has been observed that addition of metallic catalysts such as nickel (Ni) and cobalt (Co) significantly enhances the yields of biogas and methane during anaerobic digestion of animal dung [20, 25].

In the present study, we have shown the potential of non-edible resources such as weeds for the production of biofuel (bio-oil and biodiesel) through nanocatalytic gasification process by employing Co and Ni as nanocatalysts. Synthesized nanocatalysts have spherical morphology with standard crystalline structure with size distribution in the range of 20–50 nm. The chemical composition of the extracted biofuel was confirmed through Fourier transform infrared (FTIR) spectroscopy, and gas chromatography/mass spectroscopy (GC–MS) analysis. The conductivity of the byproduct (biochar) highlights the fact that obtained biochar can be used for soil remediation and an alternative to harmful chemical fertilizers which is based on organic components.

## Results and discussion

### Structural analysis of synthesized Co and Ni nanocatalysts

**Fig. 1:**
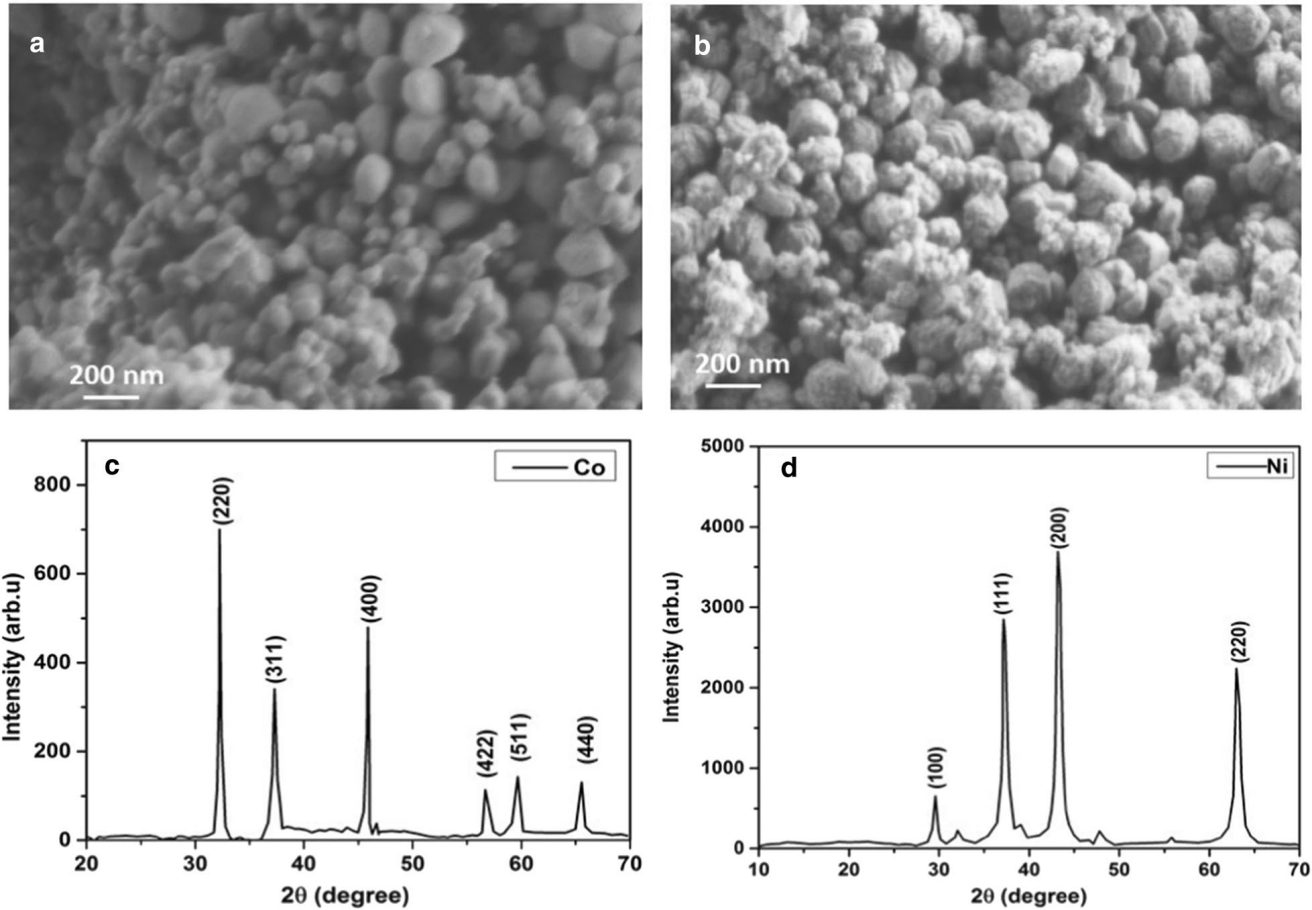
Structural analysis of nanocatalysts: **a** SEM micrograph of Co nanocatalyst; (**b**) SEM micrograph of Ni nanocatalyst; **c** XRD spectra of Co of nanocatalyst; **d** XRD spectra of Ni nanocatalysts

Surface morphology and crystal structure of the prepared nanocatalysts are shown in Fig. 1. It is clear that Co and Ni nanocatalysts have spherical morphology (Fig. 1a, b) with size distribution of 20-50 nm. The XRD results are consistent with already published work (Mahmood et al. [26] which confirms the correct crystalline phase of the Co and Ni nanocatalysts. The XRD peaks in cobalt corresponds to the indices (220), (311), (400), (422), (511) and (440) of pure phase centered cube with 2θ angles of 31.8°, 36.9°, 45.5°, 59.4° and 65.3°, respectively [27] (Fig. 1c). Figure 1d illustrates the X-ray diffracted peaks from Ni nanocatalysts which corresponds to indices (100) (111), (200) and (220) with 2θ at 29.4, 37.3, 43.4 and 62.9°, respectively [28, 29].The mean crystal sizes of the particles were calculated through Scherrer formula by calculating full width at half maximum (FWHM) of the major diffracted peaks. The estimated average crystallite size of nanocatalysts were found to be 47.92 nm and 28.85 nm Co and Ni nanocatalysts, respectively.

### FTIR analysis of *Cannabis sativa* L. biodiesel and bio-oil

**Fig. 2:**
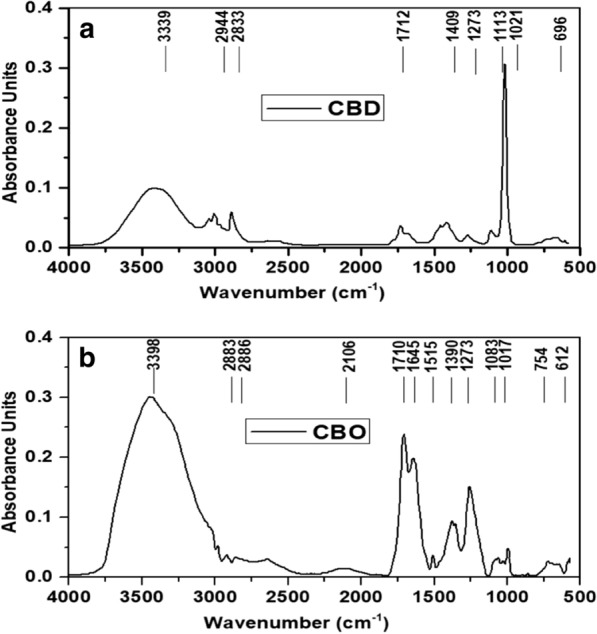
FTIR analysis of biodiesel and bio-oil: **a** Cannabis sativa L. biodiesel (CBD); **b** Cannabis sativa L. bio-oil (CBO)

Figure 2a shows the FTIR spectrum of biodiesel from *Cannabis sativa* L. with 9 major observed peaks. The FTIR spectrum indicated that first percentage absorbance peak in biodiesel was at 3339 cm^−1^ while the other was at 696 cm^−1^ whereas in bio-oil the first absorbance peak was observed at 3398 cm^−1^ and last peak was indicated at 612 cm^−1^. The observed bands between 3000 and 3700 cm^−1^ and 2700–3000 cm^−1^ show O–H and C–H bonds, respectively [30]. The observed peaks around 3398 cm^−1^, and two around 2883 cm^−1^ and 2826 cm^−1^ were observed in CBO while in CBD 3339 cm^−1^ which express the O–H stretching bonds in phenolic and alcoholic compounds. Observed bands around 2944 cm^−1^ and 2833 cm^−1^ highlight the symmetric and asymmetric stretching vibrational bands of C–H alkanes groups, respectively. In general, the broad spectrum band around 3700–3000 cm^−1^ express the OH or NH stretching vibrational band in materials with cellulose or proteins, whereas weak bands around 2924 and 2850 cm^−1^ represent CH_2_ asymmetric and symmetric stretching band, respectively [31]. It is noticed that pronounced absorption bands around 1710 cm^−1^, and 1712 cm in CBO and CBD are assigned to C=O stretch bond and suggest the presence of fatty acids in samples. The observed band around 1750–1700 cm^−1^ shows the presence of ester carbonyl group in stretching mode [32]. In CBD, the observed peaks at 1409 cm^−1^ 1515 cm^−1^ and 1390 cm^−1^ can be assigned to aromatic compounds from N–H bending mode and methyl (CH_3_) bonds. Furthermore, the intensity of 1515 cm^−1^ in CBD is substantially reduced which suggest the removal of lignin and hemicelluloses after transesterification of bio-oil. The observed bands around 1280 and 1000 cm^−1^ suggest the possible existence of acids, phenols or alcohols in the samples due to C–O vibrations [33].

### FTIR analysis of *Parthenium hysterophorus* L. biodiesel and bio-oil

**Fig. 3:**
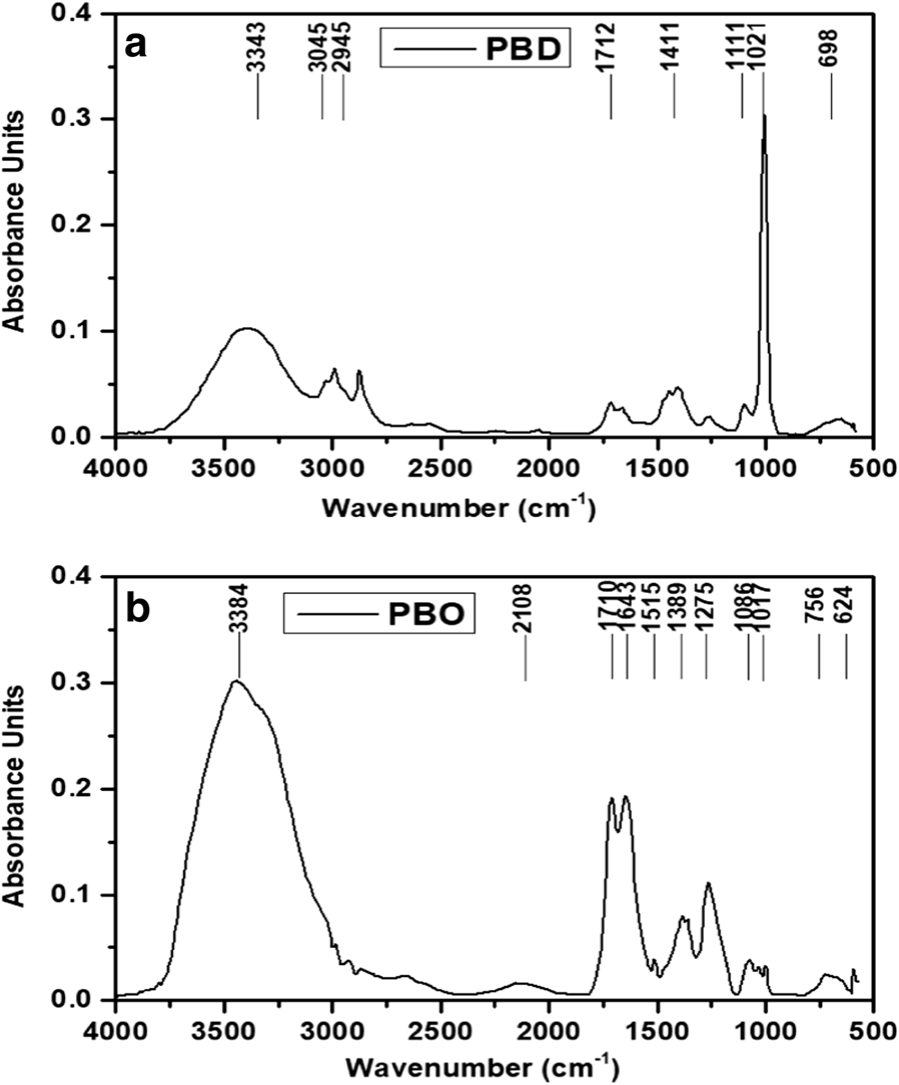
FTIR analysis of biodiesel and bio-oil: **a** *Parthenium hysterophorus* L. biodiesel (PBD); **b** *Parthenium hysterophorus* L. bio-oil (PBO)

Figure 3a shows the FTIR spectrum of biodiesel from *Parthenium hysterophorus* L. with 8 major bands. The observed peak around 3343 cm^−1^ in both PBD and PBO suggests the O–H stretching bond which can be due to the rapturing of hydrogen bonds in cellulose indicating alcohols, phenols remain unchanged in each of the blend sample which confirms the presence of above functional group in both PBD and PBO. Two peaks around 2945 cm^−1^ and 2833 cm^−1^ in PBD were detected while this band was absent in PBO. The observed peaks around 2924 cm^−1^ and 2853 cm^−1^ suggest the presence of CH_2_ and CH_3_ groups and related to symmetric and antisymmetric stretching vibrations of C–H, respectively [34]. The observed band in PBD and PBO at wave number around 1712 cm^−1^ confirms the existence of esters group with C=O stretching bond. Methyl esters also show their standard carbonyl absorptions characteristics around absorption band of 1820–1680 cm^−1^ which is absent in conventional diesel fuel [35]. Two peaks around 1643 cm^−1^ and 1515 cm^−1^ were observed in bio-oil, but were not detected in biodiesel. Our results of PBO are in good agreement with the results from Kowthaman and Varadappan [35], which confirms that slight bend in the peak around 1647 cm^−1^ ensures the absorption band of olefins. The absorbance peaks at 1389 cm^−1^ and 1275 cm^−1^ in PBD and 1409 cm^−1^ in PBO indicated alkene C–H rock, C–O stretching and alcohol O–H bending, respectively. The observed peak in PBD and PBO within the frequency band of 1120–1090 cm^−1^ confirms the presence of ester due to the stretching vibration of C–O [36]. Similar results were found during the conversion of Jatropha to biodiesel where bands around 1443, 1096 and 965 cm^−1^ disappeared and new bands were formed around 1430 cm^−1^ and 1194 cm^−1^ [36].

### GC–MS analysis of biodiesel and bio-oil from *Cannabis sativa* L.

Tables 1 and 2 summarize the results observed from GC–MS analysis of CBD and CBO showing several different chemical compounds according to their molecular weight, respectively. The color of biodiesel was yellowish and less gas was produced during gasification process. It has been noted that there are 10 and 15 major peaks in CBD and CBO, respectively. We can infer from the reported literature that main components identified in biodiesel are methyl alcohol, ethanol, trichloromethane, 2-propanone,1-hydroxy, cis-13-eicosenoic acid and methyl ester (Table 1). On the other hand, major compounds detected in the bio-oil are summarized in Table 2, which include methyl alcohol, ethanol, ethyl format, tri chloromethane, 2-propanone,1-hydroxy, 1-hydroxy-2-butanone, furfural, butyrolactone, butanoic acid, anhydride, butyric acid, p-methoxyphenyl ester, phenol, 2,6-dimethoxy, 9-Octadecenoic acid 12-hydroxy, methyl ester [z], etc. [37].

**Table 1:** GC–MS of *Cannabis sativa* L. biodiesel (CBD)

Compound name	Peak position	Molecular weight	Chemical formula
Methyl alcohol	1	32	CH_4_O
Ethanol	2	46	C_2_H_6_O
Trichloromethane	3	118	CHCL_3_
Trichloromethane	4	118	CHCL_3_
2-Propanone,1-hydroxy	5	74	C_3_H_6_O_2_
Cis-13-Eicosenoic acid, methyl ester	6	324	C_21_H_40_O_2_
Cis-13-Eicosenoic acid, methyl ester	7	324	C_21_H_40_O_2_
Cis-13-Eicosenoic acid, methyl ester	8	324	C_21_H_40_O_2_
Cis-13-Eicosenoic acid, methyl ester	9	324	C_21_H_40_O_2_
Eicosenoic acid, methyl ester	10	324	C_21_H_40_O_2_

**Table 2:** GC–MS of *Cannabis sativa* L. bio-oil (CBO)

Compound name	Peak position	Molecular weight	Chemical formula
Methyl alcohol	1	32	CH_4_O
Ethanol	2	46	C_2_H_6_O
Ethyl format	3	74	C_3_H_6_O_2_
Trichloromethane	4	118	CHCl_3_
Trichloromethane	5	118	CHCl_3_
2-Propanone,1-hydroxy	6	74	C_3_H_6_O_2_
1-Hydroxy-2-butanone	7	88	C_4_H_8_O_2_
Furfural	8	96	C_5_H_4_O_2_
Butyrolactone	9	86	C_4_H_6_O_2_
Butanoic acid, anhydride	10	158	C_8_H_14_O_3_
Butyric acid, p-methoxyphenyl ester	11	194	C_11_H_14_O_3_
Phenol, 2,6-dimethoxy	12	154	C_8_H_10_O_3_
1,2,3-Trimethoxybenzene	13	168	C_9_H_12_O_3_
Benzene, 1,2,3-trimethoxy-5-methyl-	14	182	C_10_H_14_O_3_
9-Octadecenoic acid 12-hydroxy, methyl ester [z]	15	312	C_19_H_36_O_3_

### GC–MS analysis of biodiesel and bio-oil from *Parthenium hysterophorus* L.

Same procedure was adopted for *Parthenium hysterophorus* L., and results of GC–MS from biodiesel (PBD) and bio-oil (PBO) are summarized in Tables 3 and 4, respectively. The color of the biodiesel was brown yellow liquid with less gas production during gasification process. The extracted compounds by GC–MS are summarized in Tables 3 and 4. The differences in the extracted compounds from biodiesel and bio-oil were due to the difference chemical structure of the biodiesel and bio-oil. The main extracted compounds are identified as methyl alcohol, ethanol, acetic acid, methyl ester, tri chloromethane, 2-propanone,1-hydroxy, cis-13-eicosenoic acid, methyl ester [38, 39].

**Table 3:** GC–MS analysis of *Parthenium hysterophorus* L. biodiesel (PBD)

Compound name	Peak position	Molecular weight	Chemical formula
Methyl alcohol	1	32	CH_4_O
Ethanol	2	46	C_2_H_6_O
Acetic acid, methyl ester	3	74	C_3_H_6_O_2_
Trichloromethane	4	118	CHCl_3_
2-Propanone,1-hydroxy	5	74	C_3_H_6_O_2_
Cis-13-Eicosenoic acid, methyl ester	6	324	C_21_H_40_O_2_
Cis-13-Eicosenoic acid, methyl ester	7	324	C_21_H_40_O_2_
Cis-13-Eicosenoic acid, methyl ester	8	324	C_21_H_40_O_2_
Cis-13-Eicosenoic acid, methyl ester	9	324	C_21_H_40_O_2_
Cis-13-Eicosenoic acid, methyl ester	10	324	C_21_H_40_O_2_

**Table 4:** GC–MS of *Parthenium hysterophorus* L. bio-oil (PBO)

Compound name	Peak position	Molecular weight	Chemical formula
Trichloromethane	1	118	CHCl_3_
Trichloromethane	2	118	CHCl_3_
Acetic acid	3	60	C_2_H_4_O_2_
2-Propanone,1-hydroxy	4	74	C_3_H_6_O_2_
2-Cyclopenten-1-one, 2-hydroxy-3-methyl	5	112	C_6_H_8_O_2_
6-Octadecenoic acid, methyl ester, [z]	6	296	C_19_H_36_O_2_
8-Octadecenoic acid, methyl ester	7	296	C_19_H_36_O_2_
9-Octadecenoic acid, methyl ester, [e]	8	296	C_19_H_36_O_2_
Methyl stearate	9	298	C_19_H_38_O_2_
Methyl stearate	10	298	C_19_H_38_O_2_
Octadec-9-enoic acid	12	282	C_18_H_34_O_2_
Phenol, 2,6-dimethoxy	12	154	C_8_H_10_O_3_
n-Propyl 9,12-ocatdecadienoate	13	322	C_21_H_38_O_2_
Ethyl oleate	14	310	C_20_H_38_O_2_
Ethyl oleate	15	310	C_20_H_38_O_2_

### Analysis of biochar

The biochar yield produced from Cannabis and Parthenium after catalytic gasification is 34.66% and 38.36%, respectively, which shows that biochar yield from *Parthenium hysterophorus* L. is higher as compare to *Cannabis sativa* L. The detailed analysis of the derived biochar is summarized in Table 5.

**Table 5:** Analysis of biochar

Biochar	Yield%	Organic matter%	Total organic carbon%	pH	EC (dS m^−1^)
*Cannabis sativa* L. biochar	34.66	61.75	35.82	5.3	0.4
*Parthenium hysterophorus* L. biochar	38.36	56.03	32.5	5.5	0.39

### Total organic carbon and organic matter content

The organic matter and total organic carbon compositions of biochars were determined. The total organic carbon content in *Cannabis sativa* L. was found to be 35.82%, which was higher as compare to *Parthenium hysterophorus* L. (32.5%). It is a well-known fact that high contents of carbon in biochar can lead to enhancing plant regeneration, crop production and soil health [40, 41]. In the present study, the obtained biochar from *Cannabis sativa* L. showed higher organic contents which is 61.75% in comparison to 56.03% from *Parthenium hysterophorus* L.

### pH

The pH values of the biochar indicated the least difference and showed acidic nature. It is reported that increase in temperature can increase the pH of biochars which could be due to presence of non-gasified inorganic elements in the original feedstocks [42]. In the present study, pH of 5.3 and 5.5 were recorded in the biochar of *Cannabis sativa* L. and in *Parthenium hysterophorus* L., respectively.

Kumar et al. [43] stated that the electrical conductivity (EC) and soil pH increased significantly with *Parthenium hysterophorus* L. biochar addition, but in our study, the *Parthenium hysterophorus* L. showed low pH.

### Electrical conductivity (EC)

Electrical conductivity of the soil is an important factor which highlights the presence of nutrients in the soil. Higher value of electrical conductivity leads to increase in negative charges sites, which could eventually effect the plant growth. In standard method of measurement, soil salinity indicates the ability of aqueous solution to pass current. It is very important to study the value of EC of derived biochars before the implementation to limit the deposition of salt. In our study, the electrical conductivity of 0.4 dSm^−1^ and 0.39 dSm^−1^ from biochar of *Cannabis sativa* L. and *Parthenium hysterophorus* L. has been observed, respectively, which is much lower than the saline threshold of 4 dSm^−1^ [44, 45]. Our findings suggests that derived biochar from *Cannabis sativa L* can be potential candidate which can encourage farmers to employ biochar instead chemical fertilizers which have serious concerns to environment.

### Reusability of catalyst

**Fig. 4:**
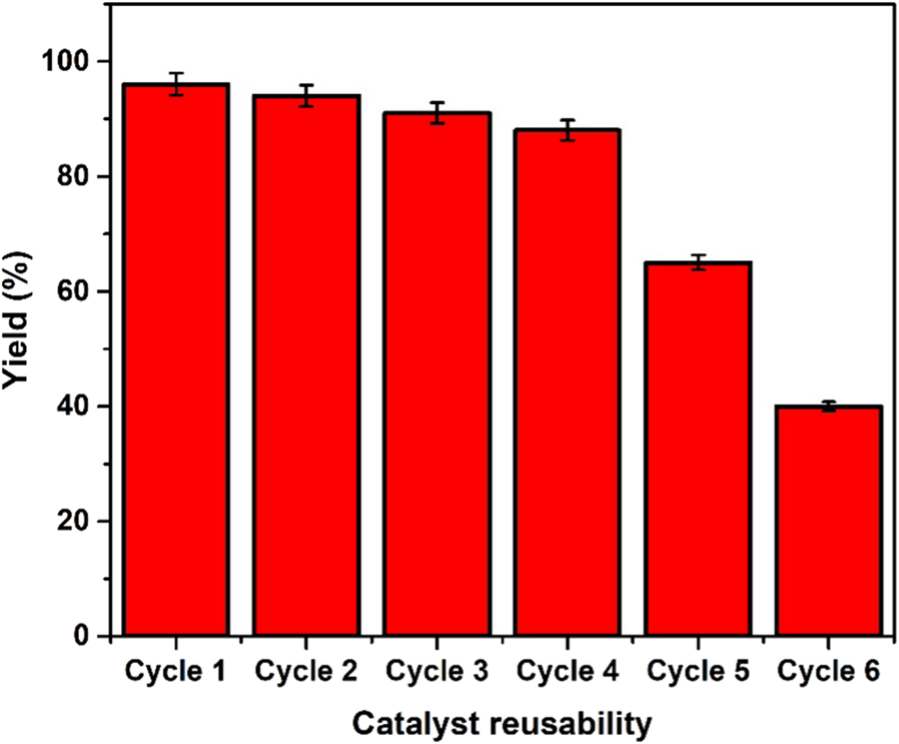
Reusability of the catalyst

The main advantage of heterogenous catalyst is its reusability as it can be easily separated from the reaction mixture. Reusability of catalysts after transesterification reaction of CBO and PBO into biodiesel was tested up to 6 cycles. At the end of each experiment, the catalysts were filtered and stirred in ethanol for 30 min to remove possible traces of polar and non-polar components present on the surface of the catalysts. The catalysts were dried at 60 °C in an oven under Ar gas flow for 12 h. It can be seen from Fig. 4 that these catalysts have good reusability up to 4 cycle, whereas efficiency dropped sharply to 65% and 40% in the 5th and 6th cycle, respectively. The decrease in catalytic activity with each cycle could due to the blockage of catalyst active sites because of the deposition of glycerol, free fatty acids and leaching of –SO_3_H from biochar [46–48].

## Conclusion

We have shown the potential of biofuel production from *Cannabis sativa* L. and *Parthenium hysterophorus L* weeds through nanocatalytic gasification which grows vastly in monsoon season. Due to high surface-to-volume ratio of nanocatalysts, the conversion of biomass to biofuel was achieved at low temperature. The results showed that efficiency of biodiesel production from *Cannabis sativa* L. is 53% which is higher as compare to available literature. The total organic carbon contents in biochar from *Cannabis sativa* L. and *Parthenium hysterophorus L* were found to be 35.82% and 32.5%, respectively. Low value of electrical conductivity of the biochar suggest that it can be implemented to reduce farmer dependence of chemical fertilizers which have serious threats to our environment.

## Materials and methods

The biomass samples of weeds namely industrial hemp (*Cannabis sativa* L.) and carrot grass (*Parthenium hysterophorus* L.) were collected from the research field area of PMAS-Arid Agriculture University Rawalpindi, Pakistan. All the samples were naturally dried and later crushed by mechanical grinder (MF 10 IKA, Werke, Germany) in submicron particle size by passing through sieve of 500μm. All the chemicals of analytical reagent grade were purchased from Sigma Aldrich. To synthesize nanocatalysts of Co and Ni, we slightly modified the procedure described in Mahmood et al. [26]. In a particular experiment, 0.5 M solution of 1, 10 phenanthroline and 0.5 M solution of cobalt chloride (CoCl_2_.6H_2_O) were individually prepared in 1-propanol. In the following step, 1,10 phenanthroline solution was added slowly into the solution of cobalt chloride with continuous stirring at 45 °C. The resultant pink precipitates were filtered and washed several times with 1-propanol to minimize the un-reacted salts. Prior to anneal samples were dried in the oven at 60 °C for 12 h to remove the moisture contents. Synthesized samples were annealed in tube furnace at temperature of 500 °C for 8 h under the flow of Ar to get the desire crystalline phase. Similar procedure was adopted to synthesize Ni nanocatalysts.

Microstructures of the synthesized Co and Ni nanocatalysts were studied by JSM-7500 scanning electron microscope (SEM) whereas crystalline structure was studied by X-ray diffractometer from PANalytical, Netherlands, (Model 3040/60 X-pert PRO).

FTIR analysis is non-destructive and the most widely employed experimental tool to analyze the chemical structure of the resulted product by studying functional groups and the bands. FTIR analysis of the transesterified biodiesel and the bio-oil obtained from studied samples was carried out by Thermo-Nicolet Nexus 670 Spectrophotometer. 1 mg of investigated sample was mixed with 100 mg of KBr to scan in the range 550 to 4000 cm^−^1 with resolution of 1 cm^−^1 [49]. All the collected gaseous samples were characterized by GC–MS Hewlett-Packard [Palo Alto, A] 5890 series II gas chromatograph with Hewlett-Packard 5972 mass selective detector by following the procedure described previously [50]. Transesterification was performed according to the methodologies adopted by Mahmood et al. [51]. The biochar was analyzed for organic matter content, total organic carbon content, pH and electrical conductivity (EC) by employing Multimeter (CRISON MM 40þ) by dissolving 1 g of biochar into 5 ml distilled water while shaking at 150 rpm for 30 min.

### Nano-catalytic gasification

In particular, experiment 100 g of respective dried biomass (*cannabis sativa* L. *and Parthenium hysterophorus* L.) was separately mixed with 1 g of nanocatalysts (Co (0.5 g) and Ni (0.5 g)) with ratio of 50/50 and gasified in a round bottom flask at 300 °C. The biochar was settled down at the of bottom flask whereas the gas was collected in gas collecting bag outside the gasifier. Furthermore, bio-oil was condensed to collect in a measuring cylinder whereas moisture contents were removed by dehydrating around 90 °C. Samples of produced gas were examined through gas chromatography–mass spectrometry. The quantity of hydrocarbon and syngas was measured. During gasification of Cannabis sativa L., we have extracted the 53.33% of oil, 34.66% of biochar and 12% gas, whereas in the case of Parthenium hysterophorus L. 44% oil, 38.36% biochar and 17.66% of gas was measured.

### Nano-catalytic transesterification

Methanol of 300 ml was mixed with 0.2 g from Ni and Co nanocatalysts with ratio of 50/50 (0.1 g Ni, and 0.1 g Co). The nanocatalyst enhanced the esterification of methanol. The solution was continuously stirred and refluxed at 80 °C for 1 h. In the next step, bio-oil samples were mixed and refluxed for 2 h at 80 °C with catalytic alcoholic mixture. The resulting mixture was allowed to settle down. Two layers with upper transparent layer of biodiesel and lower layer of used catalysts and glycerin were established. The quantity of each product was measured. The catalysts recovered by filtration were washed with ethanol to remove organic components for better performance and dried in oven at 60 °C for 12 h under Ar gas flow.

## Data Availability

Not applicable.

## Abbreviations

AAEMs: Alkali and alkaline earth metals
FTIR: Fourier transform infrared spectroscopy
SEM: Scanning electron microscope
XRD: X-ray diffraction
GC–MS: Gas chromatography/mass spectroscopy
CBD: Cannabis biodiesel
PBD: Parthenium biodiesel
CBO: Cannabis bio-oil
PBO: Parthenium bio-oil
Ni: Nickel
Co: Cobalt
EC: Electrical conductivity
NO_x_: Nitrous oxide
SO_x_: Sulphur oxide
NC: Nano-catalyst
